# THE INFLUENCE OF STAFF RETENTION ON NURSING HOME QUALITY

**DOI:** 10.1093/geroni/igz038.2578

**Published:** 2019-11-08

**Authors:** Nicholas Castle, Kathryn Hyer, John A Harris

**Affiliations:** 1 WVU, Morgantown, United States; 2 University of South Florida, Tampa, Florida, United States; 3 University of Pittsburgh, Pittsburgh, Pennsylvania, United States

## Abstract

The association of retention of Nurse Aides (NAs) with nursing home quality of care is examined. Retention is defined as staff continuously employed in the same facility for a defined period of time. Deficiency citations were used as quality indicators. Data used came from a survey of nursing home administrators, the Certification and Survey Provider Enhanced Reporting (CASPER) data, and the Area Resource File. All of the data was from 2015, and included 3,550 facilities. Analyses included negative binomial regression and multivariate logistic regression models (using GEE). The analytic modeling included staffing variables (turnover, agency use, staffing levels), facility factors (size, ownership, occupancy rate), and market characteristics (competition, Medicaid rates). The average number of deficiency citations was significantly lower (p<.01) in facilities with the higher levels of NAs consistently employed for one year or more. The same was found for facilities with the higher levels of NAs consistently employed for two years or more. While the average number of deficiency citations, the quality of care grouping of deficiency citations, and J, K, L deficiency citations were all significantly lower (p<.01) in facilities with the higher levels of NAs consistently employed for three years or more. Staff retention has been promoted as potentially influential based on little empirical evidence. The findings provide some justification for the importance of NA retention.

